# A retrospective study of *Enterococcus faecalis* infective endocarditis: comparison of clinical characteristics and outcomes associated with treatment

**DOI:** 10.1093/jacamr/dlac096

**Published:** 2022-09-30

**Authors:** N Vigneswaran, G McKew

**Affiliations:** Department of Infectious Diseases and Microbiology, Concord Repatriation General Hospital, Sydney, Australia; Department of Infectious Diseases and Microbiology, Concord Repatriation General Hospital, Sydney, Australia; University of Sydney, Sydney, Australia

## Abstract

**Introduction:**

A synergistic antibiotic combination of a penicillin and gentamicin (AG) or ceftriaxone (AC) is used in the management of *Enterococcus faecalis* infective endocarditis (EFIE). We compare the treatment outcomes between AG and AC, including low and high dose ceftriaxone (1 and 2 g 12 hourly).

**Methods:**

A retrospective cohort study of patients treated for EFIE at single tertiary centre (2012–2019). Outcome measures examined were 90- and 180-day mortality, treatment associated adverse events and relapse of bacteraemia (within 1 year).

**Results:**

39 patients were enrolled [61.6% given (AC) (*n *= 24), 24% received AC_L_ (*n *= 10) and 34% received AC_N_ (*n* = 14)], 38.4% received AG (*n *= 15). We noted a difference in the mortality outcomes at 90 and 180 days between those treated with AG and AC overall (6.7% and 33.3%, respectively) although this did not reach statistical significance (*P* = 0.114, *P* = 0.061). No significant difference was noted between these groups in incidence of relapsed bacteraemia with two cases noted in the AC cohort (8.3%, 2/24) and none observed (0/15) in the AG cohort (*P* = 0.662, *P* = 0.414). A greater number of adverse events was observed in the AG group (11/15, 73.3%) compared to the overall AC group (6/24, 25.0%) (*P* = 0.009), with no difference between the high and low dose ceftriaxone groups (*P* = 0.05).

**Conclusion:**

Combination treatment of EFIE with AC is associated with a reduced number of adverse events in comparison to AG groups. Although increased mortality was observed in the AC group, this did not reach statistical significance, and reflects the greater comorbidities and reduced capacity for surgical source control in this cohort.

## Introduction

*Enterococcus faecalis* bacteraemia is the third most common bacterium causing infective endocarditis, with most data suggesting a prevalence of 11%–26%.^1–3^*Enterococcus faecalis* infective endocarditis (EFIE) is increasing in incidence, in part due to the ageing population.^1–3^ The general principle guiding treatment of enterococcal endocarditis involves combination antibiotic therapy with a bacterial cell wall active agent (high dose penicillin or ampicillin) and a synergistically active aminoglycoside (gentamicin).^1–4^ However, more recent observational data suggests that gentamicin in this regimen can be replaced with ceftriaxone with successful clinical outcomes, irrespective of the presence of high-level aminoglycoside resistance.^5–7^ There is no randomized controlled trial data to inform these recommendations, however, over the last decade this has been incorporated into global clinical practice.^5–9^

The synergy of ceftriaxone and ampicillin or high dose penicillin in enterococcal infections been attributed to different penicillin binding proteins being the target of each drug, therefore resulting in penicillin binding protein saturation, leading to bactericidal activity.^8,9^ The dose of ceftriaxone recommended in guidelines is 2 g 12 hourly, although lower doses of 1 g 12 hourly have been noted in multiple nonrandomized studies. Our retrospective observational study aims to evaluate 8 years of clinical data to assess whether synergistic ceftriaxone dosing (at lower and higher doses) with ampicillin resulted in similar clinical outcomes to aminoglycoside combinations.

## Methods

Ethics approval was obtained on 4 June, 2020, from Sydney Local Health District Human Research Ethics Committee, Concord Repatriation General Hospital. Reference Number is CH62/6/2019-149. Given this is a retrospective audit, consent for participation and publication was waived. All methods were performed in accordance with the relevant guidelines and regulations and the Declaration of Helsinki.

We conducted a retrospective cohort study at a tertiary centre in Sydney, Australia. Patients aged 18 years and over were identified via our secure Infectious Diseases departmental blood culture and consultation database and searching terms—faecalis—enterococcal—bacteraemia—endocarditis, for the period between January 2012 and December 2019. REDCap (Research Electronic Data Capture) was used to subsequently capture and store research data.

Cases that met inclusion criteria were patients aged over 18 years diagnosed with EFIE and treated with a 4- to 6-week course of ampicillin plus gentamicin (AG) or ampicillin plus ceftriaxone (at doses of either 2 g 12 hourly or 1 g 12 hourly) (AC_N_) and without subsequent oral antimicrobial administration. Those who received lower dose ceftriaxone did so at the discretion of the Infectious Diseases physician involved. Ampicillin dosing used was 2 g 6 hourly (or therein adjusted for renal function). For AG regimens, patients required at least 2 weeks of planned gentamicin. Patients were excluded if they were treated for *E. faecalis* bacteraemia alone (instead of EFIE), treated with other antibiotic regimens for more than 7 days, were lost to follow-up or had polymicrobial bacteraemia. If therapy was subsequently altered, patients were included in the initial treatment group if subsequent antimicrobial therapy did not exceed 7 days.

The primary outcomes evaluated were 90- and 180-day all-cause mortality, all-cause in-hospital mortality and relapse of bacteraemia within 12 months; consequently, the follow-up period was over 12 months. The secondary outcomes evaluated were adverse events associated with therapy. The adverse events included nephrotoxicity, neurotoxicity, other beta-lactam toxicity, rash, vestibular dysfunction, ototoxicity or other.

Gentamicin dosing was based on synergistic dosing of 1 mg/kg 8 hourly, with trough gentamicin concentrations usually collected twice weekly, aiming at <1 mg/L. A detectable trough concentration was defined as therapeutic.

Episodes of EFIE were defined using Duke’s Criteria, or if patients were determined to have endocarditis by the treating Infectious Diseases team. Relapse of bacteraemia was defined as recurrence of blood cultures culturing *E. faecalis* within 12 months after completion of EFIE treatment.

Nephrotoxicity was defined (as per KDIGO 2012 Acute Kidney Injury Criteria) as an increase in serum Creatinine (SCr) to ≥1.5 times baseline, known or presumed to have occurred within the previous 7 days.^10^ In patients undergoing surgical intervention, SCr measured after a 5-day post-surgical period and following at least 48 hours of therapy commencement. Neurotoxicity was defined as symptoms of myoclonus or seizures, impaired consciousness, confusion, hallucinations or agitation. Beta-lactam toxicity included other adverse effects attributable to beta-lactam antimicrobials at the time of treatment, including hepatic dysfunction, leukopenia and pancytopenia. Vestibular dysfunction and/or ototoxicity was defined as symptoms of tinnitus, loss of balance, dizziness, vertigo, hearing loss and documented ototoxicity or vestibular dysfunction on serial audiometry, OAE (evoked otoacoustic emission) testing or vestibular evoked myogenic potential testing. Antimicrobial resistance is further defined to be evidence of high-level aminoglycoside resistance, noted to be *E. faecalis* isolated with gentamicin minimum inhibitory concentration ≥500 µg/L.

Fisher’s exact test was used to compare AG and AC groups for 90- and 180-day mortality, and 12-month relapse in bacteraemia. Fisher’s exact test was used to compare differences in adverse events between the groups. Differences were considered statistically significant if they had a *P* value of <0.05. Baseline demographics of each group were also collected including age, number of comorbidities, complications, whether surgery was performed, whether a prosthetic valve or intracardiac device was involved, duration of therapy and baseline renal function; Fischer’s exact test was again used to compare whether there were significant differences between the groups. Complications were defined as neurological emboli, other emboli, paravalvular abscess or new or worsening cardiac failure. Comorbidities were defined as presence of pre-existing renal impairment (both requiring and not requiring dialysis), diabetes mellitus, cirrhosis, HIV, transplant, malignancy or cardiomyopathy/cardiac failure. Statistical analyses were performed with SPSS-PC+, v.15.0 (SPSS, Chicago, IL, USA).

## Results

In total, 53 patient records were evaluated; 14 patients were excluded due to evidence of polymicrobial bacteraemia (*n *= 7), being lost to follow-up (*n *= 6) or receiving another regimen (*n *= 1). 39 patients were enrolled [62% given (AC) (*n *=* *24), 26% received low dose AC_L_ (*n *= 10) and 36% received AC_N_ (*n *= 14)]. 38% received AG (*n *= 15).

### Demographics and baseline characteristics

The demographics and baseline characteristics of the patients included in the final analysis are detailed in Table 1.

**Table 1.** Demographics of included patients

	Ampicillin + Ceftriaxone 1 g (*n* = 10)	Ampicillin + Ceftriaxone 2 g (*n* = 14)	Ampicillin + Gentamicin (*n* = 15)	Significance (*P* value <0.05)
Age, median (IQR)	80 (15)	84.5 (6)	79 (13)	0.431
Number of comorbidities, median (IQR)	2.5 (1)	3 (1)	2 (1)	0.703
Patients with complications, number (proportion)	9 (0.9)	11 (0.79)	14 (0.93)	0.502
Surgery performed, number (proportion)	0 (0)	0 (0)	5 (0.33)	0.014
Vegetation—site				
Left	6 (0.6)	7 (0.5)	9 (0.6)	
Right	4 (0.4)	6 (0.43)	4 (0.26)	
Unknown	0	0	1 (0.07)	
Device-related	0 (0)	1 (0.07)	1 (0.07)	
Prosthetic valve, number (proportion)	7 (0.7)	9 (0.64)	10 (0.66)	0.958
Isntracardiac devices	2 (0.42)	2 (0.42)	3 (0.07)	0.7
Gentamicin therapeutic drug monitoring performed, number (proportion)	0 (0)	0 (0)	15	
Duration of therapy, median (IQR)	43 (2)	42 (2)	41 (3)	0.3
Renal function at commencement, median (IQR)	133.5 (55)	108.5 (70)	98 (75)	0.234

There was no statistically significant difference in number of comorbidities (*P* = 0.703) or complications (*P* = 0.502) of IE between the three groups. There was a statistically significant difference in the surgeries performed between the groups (*P* = 0.014), with no cases of surgery performed in the AC group, but five cases in the AG group. There was no statistically significant difference in the presence of prosthetic valve or intracardiac devices (*P* = 0.958, and *P* = 0.7) or renal function (SCr in µM/L) at commencement of therapy (*P* = 0.234). The median duration of therapy was not statistically different between the groups (*P* = 0.3), with the median duration of therapy in days for the AG and AC groups, 42 and 43 days, respectively.

In the cases where surgery was performed (in the AG group), they were due to persistent bacteraemia, vegetation size in two cases, paravalvular abscess and heart failure with severe valvular regurgitation. Additionally, surgery was indicated but not performed in 5/39 (12.8%) patients in the AC group.

There were three cases of high-level aminoglycoside resistance in the AC group.

### Primary outcome measures

The mortality of all study patients included with EFIE was 20.5% at 90 days, with mortality of 33.3% and 6.7% in the AC and AG groups, respectively; the mortality figures were 30%, 28.6% and 6.7% for the AC_L,_ AC_N_ and AG groups, respectively. The mortality of all study patients included with EFIE was 23.1% at 180 days, with mortality of 40%, 28.6% and 6.7% for the AC_L,_ AC_N_ and AG, respectively. The mortality outcomes at 90 and 180 days between: (i) those treated with a gentamicin combination and ceftriaxone combination overall (*P* = 0.114, *P* = 0.061) and (ii) between high and low dose ceftriaxone (*P* = 1.0, *P* = 0.673) did not reach statistical significance. These figures are listed in Table 2. Only two cases of relapsed bacteraemia were noted, in cases of patients treated with AC (2/24, 8.3%) with none observed in AG (0/15, 0%); in both cases, the cause was linked to comorbidities and inability to hence achieve surgical source control. The differences between AC and AG groups in terms of relapsed bacteraemia did not reach statistical significance (*P* = 0.662, *P* = 0.414). Four of the patients in the AC group in whom surgery was indicated and not performed, died within a 180-day follow-up period; there was therefore an 80% (4/5) all-cause 180-day mortality observed in this subset.

**Table 2.** Mortality 90 and 180-days

		Therapy		
	Total	Ampicillin + Ceftriax one 1 g	Ampicillin + Ceftriax one 2 g	Ampicillin + Gentamic in
				
Total	39	10	14	15
Dead	9	4	4	1
	23.1%	40.0%	28.6%	6.7%
Alive	30	6	10	14
	76.9%	60.0%	71.4%	93.3%

### Secondary outcome measures

A greater number of adverse events was observed in the AG group (11/15, 73.3%) compared to the AC group (6/24, 25.0%) overall (*P* = 0.009), with no difference between the high and low dose ceftriaxone groups (*P* = 0.051). The adverse events are listed in Tables 3 and 4.

**Table 3.** Occurrence of adverse event

		Therapy		
	Total	Ampicillin + Ceftriax one 1 g	Ampicillin + Ceftriax one 2 g	Ampicillin + Gentamic in
				
Total	39	10	14	15
Yes	17	5	1	11
	43.6%	50.0%	7.1%	73.3%
No	22	5	13	4
	56.4%	50.0%	92.9%	26.7%

**Table 4.** Type of adverse event

		Therapy		
	Total	Ampicillin + Ceftriax one 1 g	Ampicillin + Ceftriax one 2 g	Ampicillin + Gentamic in
Had an adverse event	17 (43.1%)	5 (12.8%)	1 (2.6%)	11 (28.2%)
Rash	1	1	—	—
Beta-lactam toxicity	1	1	—	—
Nephrotoxicity	9	1	—	8
Neurotoxicity	2	1	1	—
Vestibular dysfunction or ototoxicity	3	—	—	3
Other	1	1	—	—

Of the total nine patients who experienced nephrotoxicity, 5/9 patients had progressed to new chronic kidney disease as per KDIGO 2012 criteria of GFR <60 mL/min/1.73 m^2^ (GFR categories G3a–G5) for >3 months at 90 days and then 180 days follow-up.^10^ The other four achieved recovery of their renal function at 90- and 180-day follow-up. The SCr of the patients at commencement, discharge, 90- and 180-day follow-up is detailed in Table 5.

**Table 5.** Breakdown of renal function in patients who had nephrotoxicity

Patient with nephrotoxicity	Assigned antimicrobial regimen	SCr (μmol/L) and eGFR (mL/min/1.73 m^2^) at commencement of therapy	SCr (μmol/L) and eGFR (mL/min/1.73 m^2^) at discharge	SCr (μmol/L) and eGFR (mL/min/1.73 m^2^) at 90-day follow-up	SCr (μmol/L) and eGFR (mL/min/1.73 m^2^) at 180-day follow-up
1	AG	90 eGFR 71	Cr 138 eGFR 50	110 eGFR 55	109 eGFR 55
2	AG	88 eGFR 75	Cr 135 eGFR 50	115 eGFR 56	119 eGFR 53
3	AG	82 eGFR 80	CR 129 eGFR 52	Cr 120 eGFR 49	Cr 122 eGFR 48
4	AG	76 eGFR 72	Cr 115 eGFR 57	Cr 100 eGFR 58	Cr 110 eGFR 56
5	AG	100 eGFR 67	Cr 152 eGFR 40	Cr 135 eGFR 45	Cr 130 eGFR 48
6	AG	102 eGFR 69	Cr 155 eGFR 38	Cr 100 eGFR 70	Cr 105 eGFR 68
7	AG	105 eGFR 68	Cr 160 eGFR 37	Cr 110 eGFR 65	Cr 99 eGFR 68
8	AG	99 eGFR 72	Cr 152 eGFR 36	Cr 90 eGFR 76	Cr 93 eGFR 74
9	ACL	Cr 138 eGFR 42	Cr 210 eGFR 25	Cr 142 eGFR 40	Cr 137 eGFR 41

In four cases of the AC group (all given AC_L_), adverse events necessitated an interruption to antimicrobials, in three out of four cases (on days 14, 17 and 21 of treatment, respectively) for a period of 48, 72 and 72 hours, respectively; in the other case, ceftriaxone therapy was interrupted for a 72-hour period and recommenced. In five cases of the AG group, adverse events necessitated cessation of gentamicin (on days 10, 12, 14, 15 and 22 of treatment), with no other antimicrobial added.

## Discussion

Our findings are in keeping with previous studies demonstrating a higher incidence of adverse events in those treated with an aminoglycoside combination in comparison to ceftriaxone combinations.^6,7,9,11–17^ Although it did not reach statistical significance, our study did demonstrate higher all-cause mortality, in-hospital death and rates of relapse between patients treated with ceftriaxone than in aminoglycoside-based regimens. This is probably reflective of our smaller sample size and is influenced by the younger median age of those treated with gentamicin-based regimens.

There was no significant difference between all groups in the demographic measures of age, comorbidities, baseline renal function, complications of IE or presence of prosthetic device. However, a significantly greater number of cardiac and extracardiac surgeries were performed during the treatment course of EFIE for patients treated with AG, again in the setting of the younger age of this cohort (though this was not statistically significant). We importantly note that 50% of the group who died (4/8) in the AC group had indications for surgery that was not performed in the setting of increased comorbidities and surgical risk; this probably confounded their increased 180-day all-cause mortality. Despite the limitations in sample size and statistical power, our study findings correlate findings in previous studies suggesting a frailer and more comorbid cohort that receives ceftriaxone combination therapy in favour of aminoglycosides.^11–18^ This is also reflective of real-world clinical practice.^12–14^ We would advocate that ceftriaxone (AC) combination therapy should still be used given its lower rates of adverse events, clinical experience and existent data to support its use.^6,7,11–18^

A recent study evaluating outpatient EFIE treatment with continuous benzylpenicillin infusion in combination with once daily aminoglycoside or ceftriaxone, found no difference in treatment success between the groups, and included patients who had been treated with low and high dose ceftriaxone (2 g once daily or 1 g 12 hourly).^11^

The adverse event most associated with aminoglycoside therapy within our study, was nephrotoxicity, reflecting the findings of previous studies. Only one case of nephrotoxicity was noted in our study in the AC group; eight cases were noted in the AG group (53%). In keeping with the recommendations in previous studies, we would advocate for consideration of ceftriaxone based dual beta-lactam therapy to be initiated in cases of patients with or at risk of renal impairment with EFIE.^6,7,11–18^

The alternative option suggested, to reduce toxicity and treatment related adverse events, is a shorter course of therapy in both AG and AC regimens. Two studies have examined shorter course (4 weeks as opposed to 6 weeks) AC regimens in treating *E. faecalis* endocarditis, one of which found the shorter course regimen was associated with a higher rate of relapse.^17,18^ Multiple Danish studies demonstrated efficacy in clinical cure, mortality and incidence of relapse with a 6 week ampicillin and 2 week aminoglycoside regimen for EFIE, the first of which resulted in a change to the Danish Society of Cardiology recommendations for aminoglycoside therapy duration in EFIE.^19,20^

Many centres use outpatient parenteral therapy programmes to facilitate ongoing administration of either AG or AC regimens in EFIE. Herrera Hidalgo *et al.* recently demonstrated the administration of a single daily dose of 4 g of ceftriaxone with ampicillin-based outpatient regimens had a higher rate of relapse in comparison to a 12 hourly 2-g dose of ceftriaxone.^21^ Two other small retrospective case series have demonstrated the efficacy of EFIE treatment with outpatient penicillin infusions in combination with ceftriaxone at 2 g 12 hourly dosing at achieving clinical cure.^22,23^

Our study also did not note a significant difference in adverse events between those treated with low- and higher-dose ceftriaxone regimens. Beta-lactam toxicity, particularly in the elderly, has been linked to altered pharmacokinetics due to multifactorial mechanisms including reduced renal clearance, and the pathophysiological changes associated with critical illness.^23,24^ Theoretically, modified or reduced dosing could lower the risk of development of beta-lactam related toxicity.^23,24^ However, most pharmacokinetic studies propose altered dosing be guided by therapeutic drug monitoring (i.e. with ceftriaxone levels) in at risk or elderly patients.^23,24^

### Limitations and future directions

There were several limitations to our study, mainly the small sample size, the implications of the statistical power of our findings and retrospective cohort design. This was not a randomized or prospective study, and no intention to treat analysis was performed. The significant differences in the demographic measures of surgeries performed and duration of treatment should also be interpreted in the context of small numbers of patients evaluated in each of the antimicrobial regimen cohorts. There is a confounding bias in the number of patients within the ceftriaxone cohort who were eligible for surgery but could not receive it that probably contributed to the increased mortality in this cohort in comparison to the gentamicin subset. A selection bias could be introduced by assigning patients to receive AC or AG at physician discretion that may contribute to older more comorbid patients receiving AC over AG. Excluding patients who were lost to follow-up, or who had different antibiotic regimens for more than 7 days, could also have contributed to selection bias.

therapeutic drug monitoring was not assessed in our study for ceftriaxone. Trough gentamicin concentrations, although collected, were not always correlated with clinical evidence of gentamicin toxicity such as ototoxicity or nephrotoxicity. Although a standard 1 mg/kg, three times daily gentamicin dose was used, there was some variability, at the discretion of the treating physician, in dosing for obese patients.

Exploration of alternative therapies is required, particularly regarding logistics, toxicity and clinical cure in outpatient settings as well as evaluation of early transition to oral agents.^25,26,27^ Oral agents proposed in the POET trial included combinations of amoxicillin, rifampicin, moxifloxacin and linezolid.^27^ A case report has also noted successful treatment of EFIE with oral amoxicillin-clavulanate.^28^

Further randomized controlled data, with evaluation of therapeutic drug monitoring of beta-lactam antimicrobials, is needed to guide recommendations around lower dose ceftriaxone in EFIE. Studies that incorporate correlations of hypoalbuminemia and ceftriaxone levels in EFIE should also be explored, given the known concentration-dependent albumin binding of ceftriaxone.

### Conclusion

Our findings support existing data, advocating for use of AC regimens in EFIE, particularly in patients with or at risk of aminoglycoside toxicity such as renal impairment, given the higher rates of adverse events were associated with aminoglycoside therapy. Although increased mortality was observed in the AC group, this did not reach statistical significance and reflects the increased frailty, greater comorbidities and reduced capacity for surgical source control in this cohort. Given the non-significant differences in mortality between the AC and AG regimens, larger randomized control trials are recommended. Further data incorporating therapeutic drug monitoring of ceftriaxone and larger randomized control clinical trials are required.

## Data Availability

The datasets generated during and/or analysed during the current study are available from the corresponding author on reasonable request.
